# Preliminary Study on Biosensor-Type Time-Temperature Integrator for Intelligent Food Packaging

**DOI:** 10.3390/s18061949

**Published:** 2018-06-15

**Authors:** A. T. M. Mijanur Rahman, Do Hyeon Kim, Han Dong Jang, Jung Hwa Yang, Seung Ju Lee

**Affiliations:** Center for Intelligent Agro-Food Packaging (CIFP), Department of Food Science and Biotechnology, Dongguk University, Seoul 10326, Korea; mijananftiubd@gmail.com (A.T.M.M.R.); goreh1@naver.com (D.H.K.); gksehd7609@naver.com (H.D.J.); alsdn4547@naver.com (J.H.Y.)

**Keywords:** TTI, biosensor, glucose oxidase, electrochemical reaction, intelligent food packaging

## Abstract

A glucose biosensor was utilized as a platform for the time-temperature integrator (TTI), a device for intelligent food packaging. The TTI system is composed of glucose oxidase, glucose, a pH indicator, and a three-electrode potentiostat, which produces an electrical signal as well as color development. The reaction kinetics of these response variables were analyzed under isothermal conditions. The reaction rates of the electrical current and color changes were 0.0360 ± 0.0020 (95% confidence limit), 0.0566 ± 0.0026, 0.0716 ± 0.0024, 0.1073 ± 0.0028 µA/min, and 0.0187 ± 0.0005, 0.0293 ± 0.0018, 0.0363 ± 0.0012, 0.0540 ± 0.0019 1/min, at 5, 15, 25, and 35 °C, respectively. The Arrhenius activation energy of the current reaction (*E*a_current_) was 25.0 ± 1.6 kJ/mol and the *E*a_color_ of the color reactions was 24.2 ± 0.6 kJ/mol. The similarity of these *E*a shows agreement in the prediction of food qualities between the electrical signal and color development. Consequently, the function of the new time-temperature integrator system could be extended to that of a biosensor compatible with any electrical utilization equipment.

## 1. Introduction

In the course of modernization, there has been a global expansion of food distribution systems. Because the quality and safety of chilled and frozen foods are strongly influenced by temperature, the monitoring of time-temperature history throughout the entire distribution chain is necessary [1]. Commercially available monitoring devices include temperature data loggers, time-temperature integrators (TTIs), smart radio frequency identification (smart-RFID), and others. The TTI is a colorimetric label attached to food packages, which exhibits visual color changes depending on the time-temperature history, predicting the food quality status. TTIs offer several advantages over other devices, including small size, low cost, and easy operation. The TTI color change is based on physical, chemical, or biological reactions [2,3], depending on temperature [4]. The performance of TTIs for monitoring food deterioration due to the temperature abuse during the storage or distribution has been successful in many cases such as kimchi [5], frozen vegetables [6,7], fish products [8,9,10,11], meat products [12,13,14], mushrooms [15], and dairy products [16,17].

Unfortunately, despite longstanding recognition of TTIs as effective monitoring tools, their commercialization is still in the beginning stages. There are multiple limitations associated with time-temperature integrators such as legislative rules, accuracy, and quality indication by visible color change, among others [18,19], which are barriers to widespread adoption of this technology in cold-chain monitoring. In particular, decision-making based only on the visual observation of TTI color can be confusing; this hinders adoption by the food industries and acceptance by consumers. If TTI responses could be converted to digital information, that would address the problems associated with interpreting visible colorimetric information. For example, glucose-detecting redox-enzyme-based biosensors can produce an electrical current on interaction with glucose; others can change color. This raises the possibility that even a colorimetric TTI could become a biosensor-type TTI (biosenTTI). Further, information technology (IT) is being applied to monitoring, logistics, and other aspects of food distribution systems [20,21]. The use of biosenTTI devices could be extended to IT-based food package logistics.

A biosensor is an analytical device with a bio-recognition element that converts the biological response into a measurable signal, often electrical, which may provide direct information. The development of various classes of biosensors based on different operating principles electrochemical (amperometric, potentiometric) [22], optical [23], piezoelectric [24], and others [25,26] has allowed their application for analyzing a wide range of substances. Even though biosensor technology was developed almost 60 years ago, there are several applications that are still to be studied. The application of biosensor technology to TTI is an example [27]. described a TTI based on an amperometric glucose oxidase (GOx) biosensor that was the first and, until now, the only one studied in the field of biosenTTI. This is a specialized TTI: a lethality indicator appropriate for use in food sterilization, rather than general food package storage or distribution. This TTI was evaluated for the kinetics of thermal inactivation of the immobilized enzyme under isothermal conditions between 70 and 79.7 °C as well as current generation in order to evaluate the potential of the proposed biosenTTI for food pasteurization processes. However, a biosenTTI for monitoring the quality of chilled foods during storage and transportation has not yet been attempted. 

In this study, the GOx biosenTTI used for pasteurization was adapted for use at chilled temperatures. We assumed that the TTI response depends on the temperature sensitivity of the GOx redox reaction in the range of storage or distribution temperatures. The biosenTTI system was composed of a recognition element with GOx, a redox substrate and colorimetric indicator, and a transducing element with a potentiostat. The electrochemistry of the system was analyzed to find the optimum input voltage of the potentiostat. The reaction kinetics and Arrhenius temperature dependency were identified. The electrical signals were also correlated with the TTI color responses. 

## 2. Materials and Methods

### 2.1. Materials

Glucose oxidase (EC 1.1.3.4, type X-S from Aspergillus niger, 117200 U/G solid), glucose, methyl red sodium salt and dextrose anhydrous were purchased from Sigma-Aldrich (St. Louis, MO, USA). Deionized ultra-filtered water (Ω > 18 MΩ.cm, Fisher scientific, Pittsburgh, PA, USA).

### 2.2. Creation of Biosensor-Type TTI 

**Figure 1:**
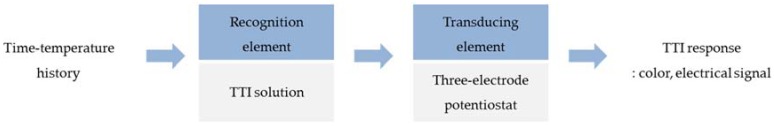
Construction of the biosensor system with time-temperature integrator (TTI) function.

The TTI was based on a glucose biosensor platform [27]. The TTI system was composed of TTI solution and a potentiostat system (Figure 1). The GOx solution of 0.0015 g/mL or 175.8 U/mL was prepared using 2 mM sodium acetate (pH 5.5). The substrate concentration was determined by identifying K_m_ value.

(1) $$V_{0}=V_{max}\frac{\left[S\right]}{\left[S\right]+K_{m}},$$

(2) $$K_{m}=\frac{k_{-1}+k_{2}}{k_{1}}.$$

Equation (1) is re-written as the Lineweaver-Burk plot used in regression analysis.

(3) $$\frac{1}{V_{0}}=\frac{K_{m}}{V_{max}}\cdot \frac{1}{S}+\frac{1}{V_{max}}.$$

To make a final concentration of the substrate solution, a stock glucose solution (1.0 M) was diluted.

The TTI solution was activated by mixing the enzyme solution, substrate solution, and pH indicator of methyl red sodium salt (0.0002 g/mL). Concentrations of the GOx solution were 20 µL and 160 µL, and those of the glucose solution were 3 mL and 10 mL for color and electrical signal measurements, respectively.

The potentiostat system (VSP-300, Biologic Science Instruments, Paris, France) was used to measure the TTI electrical signal. Three electrodes (glassy carbon, Ag/AgCl, and platinum wire) were immersed in the TTI solution contained in a 20 mL cell vial. Prior to use, the glassy carbon electrode was polished with 0.05 µm alumina slurry, followed by washing in deionized ultra-filtered water and allowed to dry at room temperature.

### 2.3. Color Measurement of TTI

The color of the TTI solution was measured by a spectrophotometer (UV-1800 PC, Shimadzu Co., Kyoto, Japan); 3 mL of the TTI solution was used. The absorbance spectra of the samples were scanned from 400–700 nm to find the optimal wavelength for measurement. When there was an absorbance saturation, the solution was diluted and the absorbance was corrected according to the dilution ratios.

### 2.4. Electrical Signal Measurement of TTI

A linear sweep voltammogram was first performed in the voltage range of 0.1 to 1.5 V at the scan rate of 50 mV/s, and an optimal voltage was selected for the current measurement as the electrical signal of TTI. 

### 2.5. Determination of Kinetic and Arrhenius Parameters of TTI Responses

The activated TTI solutions (30 mL each) were stored in an incubator at different temperatures (5, 15, 25, and 35 °C), in beakers covered with plastic film. At certain intervals, the solutions were removed from the incubator and used to measure color change and electrical response. Then, they were returned to the incubator and used for the next measurements.

The rates of color and electrical current changes of TTI were estimated from the data under isothermal conditions. Then, the Arrhenius activation energy (*E*a) was estimated from the rates.

The color and current variables could be expressed as follows:

Y = −*kt*, (4)

where Y is either the color or current variable, *k* is the rate constant of reaction or the variable change, and *t* is the reaction time.

The activation energy (*E*a) was calculated by taking the natural logarithm on both sides of the Arrhenius equation.

(5) $$\ln k=\frac{Ea}{RT}+\ln\;k_{0},$$

where *E*a, *T*, *R*, and *k*_0_ are the Arrhenius activation energy (kJ/mol), the absolute temperature (K), the gas constant (8.314 × 10^−3^ kJ/mol K), and the pre-exponential factor, respectively. 

### 2.6. Statistical Analysis

The color and current measurements were repeated 5 and 3 times, respectively. More repetition on color measurement was due to environmental variables such as light. The averages of color or current were used in linear regression analysis for *k* estimation [28]. The *k* was ultimately used in another regression analysis for *E*a estimation. Actually, the *k* from averages and total original values are statistically the same, resulting in the same *E*a. A Microsoft Excel program was used.

## 3. Results and Discussion

### 3.1. Characterization of BiosenTTI Responses

Conventional TTI is an indicator, whose response is a color change showing the status of packaged food qualities. The biosenTTI was devised to respond electrically as well as colorimetrically. The mechanism of the biosenTTI is D-gluconic acid and H_2_O_2_ production as a result of a reaction between GOx and β-D-glucose. As the D-gluconic acid donates H^+^ in the solution, the pH of the solution drops. This acidic property of D-gluconic acid was used to measure the color development of TTI by using a suitable pH indicator. This color change reflects the deterioration status of food products.

**Figure 2:**
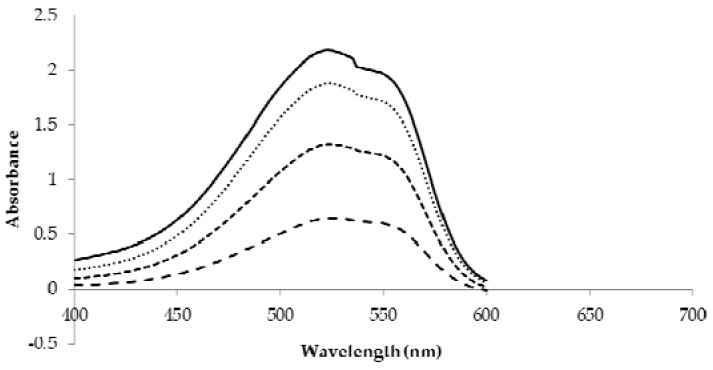
Measured absorption spectra of the TTI solution at different concentrations. “—”: measured by 40 mM glucose and GOx, ⋅ ⋅ ⋅ ⋅ ⋅: 30 mM glucose and GOx, - - - - - : 20 mM glucose and GOx, – – – – : 10 mM glucose and GOx.

Figure 2 shows the peaks corresponding to the D-gluconic acid produced by the reaction between GOx and glucose solution with a red sodium salt pH indicator. Methyl red sodium salt becomes red under pH 4.4 and yellow over pH 6.2. GOx catalyzes the oxidation of β-D-glucose to produce one molecule of D-gluconic acid and H_2_O_2_. The product D-gluconic acid dissociates in solution and releases hydrogen ion. Therefore, D-gluconic acid changes the pH of the solution and consequently changes the color of the entire solution from yellow to red. The wavelength at which maximum absorption was observed, 523 nm, was employed throughout the experiments.

**Figure 3:**
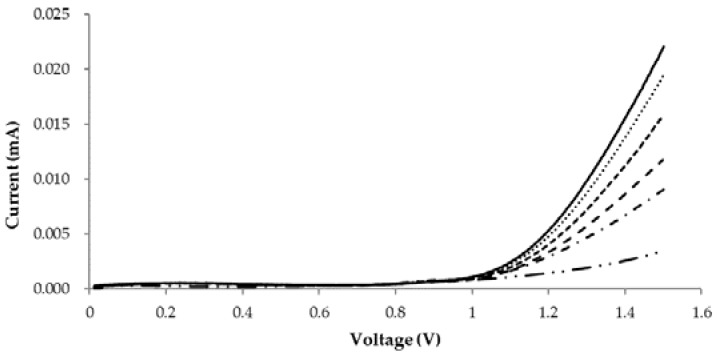
Plot of the current generation against applied potential measured by linear sweep voltammetric method after storage TTI solution at 25 °C with certain interval time. —: 80 min, ⋅ ⋅ ⋅ ⋅ ⋅: 60 min, - - - - - : 40 min, – – – –: 20 min, –⋅–⋅–⋅–⋅: 10 min, and –⋅⋅–⋅⋅–⋅⋅: only glucose solution.

Figure 3 shows the current measured by the linear sweep voltammetric method using a potentiostat, corresponding to the H_2_O_2_ production. In the GOx-catalyzed redox reaction, the reaction is represented by the following reaction equations [29].

GOx (FAD) + 2e^−^ + 2H^+^ → GOx (FADH_2_), (6)

GOx (FADH_2_) + O_2_ → GOx (FAD) + H_2_O_2_, (7)

where FAD is flavin adenine dinucleotide. The produced H_2_O_2_ can be electrochemically oxidized to oxygen at a sufficient positive electrode potential at the working electrode of the potentiostat, releasing the electrons and protons that were originally on glucose. In Figure 3, the X-axis represents the input voltage, while the Y-axis represents the response of the TTI expressed as current. The current generated at +1.3 V was found to change linearly with reaction time at all temperatures. At the other input voltages, the changes were not linear (data not provided in this report), and therefore the voltage of +1.3 V was chosen to read the current as TTI responses.

**Figure 4:**
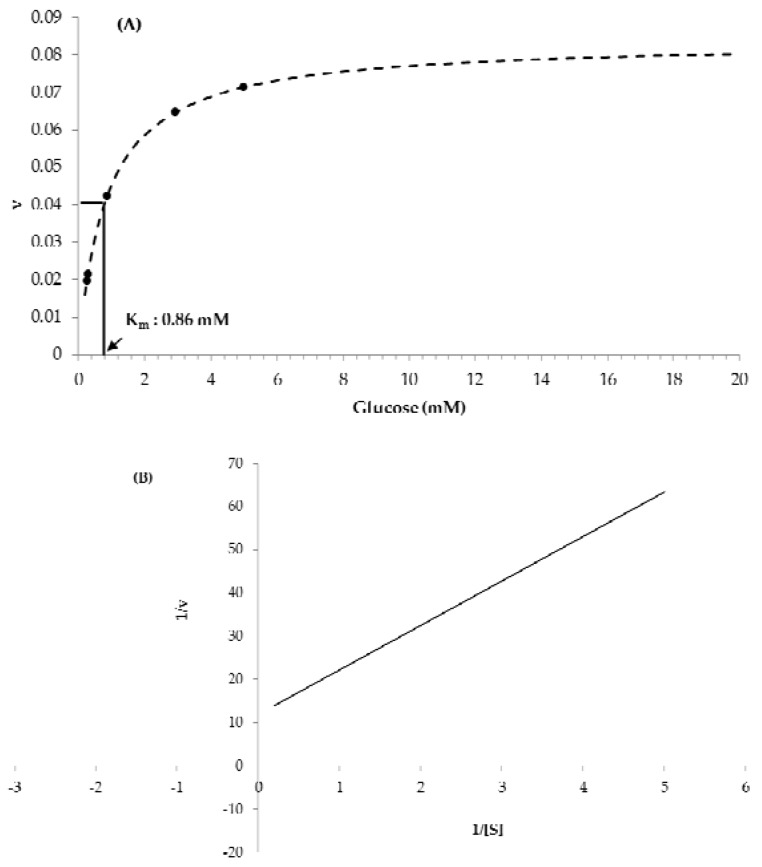
(**A**) Plot of the reaction velocities against glucose concentrations (mM) and (**B**) Lineweaver-Burk plot of the experimental data.

The Michaelis constant, K_m_, was evaluated to determine the substrate concentration sufficient to maintain a zero-order reaction. Figure 4 shows the dependence of reaction rate on different concentrations of glucose substrate. From the Lineweaver-Burk plot [30], the K_m_ value of GOx was estimated to be 0.86 mM, indicating a very high affinity for glucose. The final glucose concentration was determined to be 10 mM, ten times higher than the K_m_. In order to formulate the TTI composition, the concentration of substrate should be at least 10 times higher than the K_m_ value so that the enzyme is fully saturated by the substrate, consistent with zero-order kinetics [31]. Otherwise, there is a possibility of substrate inhibition and the zero-order kinetics will be transformed to first-order (or a higher order) kinetics; in that case, an estimation of the exact shelf-life of foods would not be possible.

### 3.2. Kinetics and Temperature Dependency of BiosenTTI 

**Figure 5:**
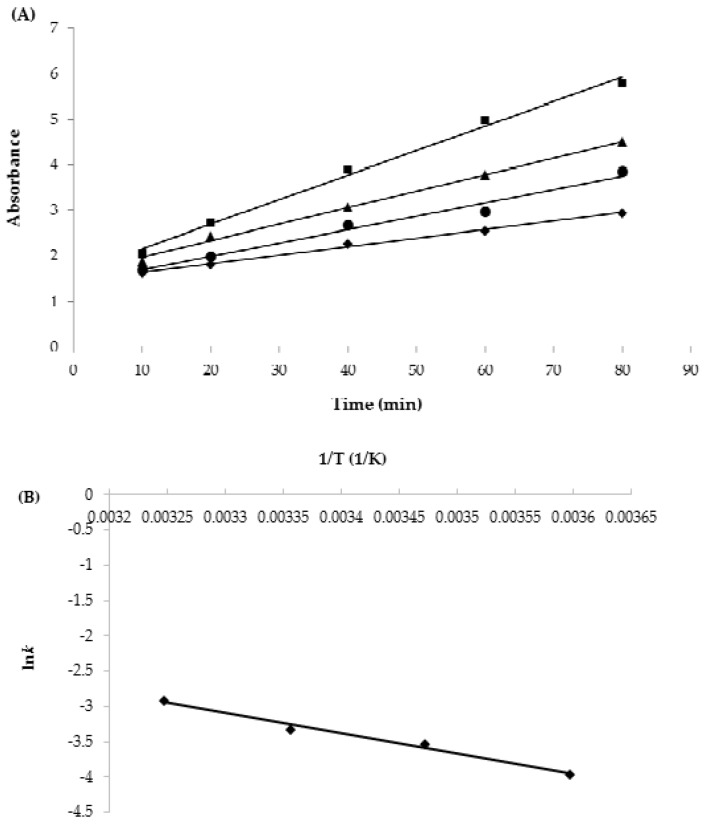
(**A**) Plot of color change at different isothermal temperatures (◆: 5 °C, ●: 15 °C, ▲: 25 °C, ■: 35 °C) and (**B**) Arrhenius plot of the reaction rate constants (abs) against temperature.

The color and the rate of color change increased with the time and temperature, respectively (Figure 5). The color change with time belonged to a zero-order reaction, having high coefficients of determination (R^2^) ranging from 0.98–0.99 (Table 1).

The zero-order enzyme reactions could be obtained by high concentrations of glucose substrate. If there were not enough glucose, substrate inhibition would lead to an inconsistent reaction order higher than zero. The temperature dependence of the reaction rates affected the Arrhenius relations as well, having an R^2^ of 0.99. The *E*a_color_ was estimated to be 24.2 ± 0.6 kJ/mol. This *E*a_color_ value is too low to monitor the quality of most foods, which have an *E*a much higher than this. However, in our previous work using a laccase-based TTI, the *E*a of another redox enzyme that was also very low was successfully increased. In a previous study, the *E*a was increased and regulated by adding sodium azide, a competitive inhibitor of laccase, or by making a mixture of isoenzymes with a different activation energy [32,33]. Therefore, the low *E*a_color_ of the GOx-based TTI could be increased. 

**Figure 6:**
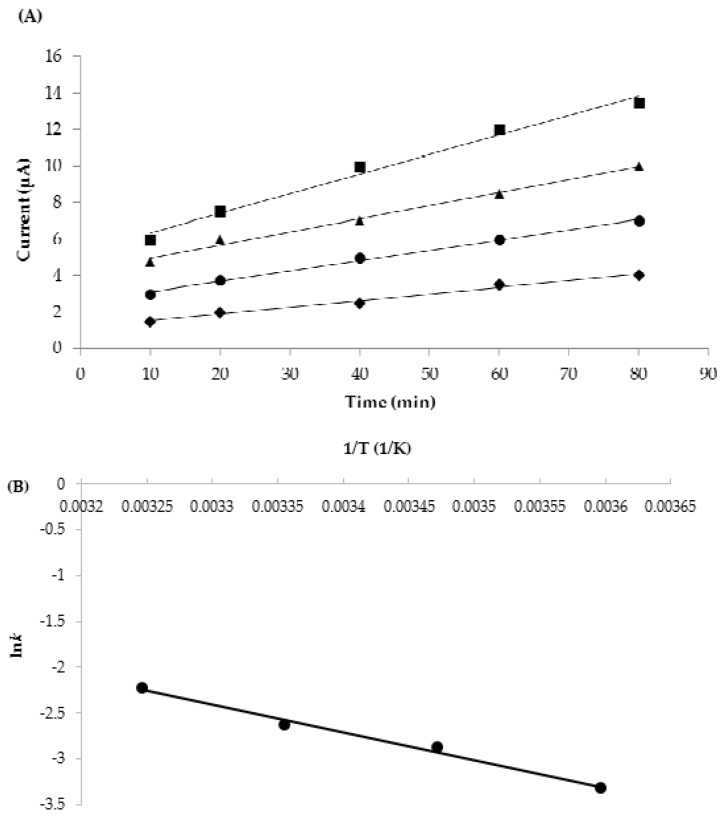
(**A**) Plot of current generation at different isothermal temperatures (◆: 5 °C, ●: 15 °C, ▲: 25 °C, ■: 35 °C) against time (min) at +1.3 V and (**B**) Arrhenius plot of the reaction rate constants (current) against time.

Figure 6 shows the current generated at +1.3 V. The current generation increased linearly with time, indicating a zero-order reaction, and the reaction rates increased with temperature. The reaction rates of the current changes are 0.0360 ± 0.0020, 0.0566 ± 0.0026, 0.0716 ± 0.0024, and 0.1073 ± 0.0028 μA/min at 5, 15, 25, and 35 °C, respectively (Table 1). The relationship between ln*k* against 1/T was almost linear and *E*a_current_ was estimated as 25.0 ± 1.6 kJ/mol, having an R^2^ of 0.99. This *E*a_current_ is almost the same as that of the color change of TTI (24.2 ± 0.6 kJ/mol). This is because the color change with pH decrease and the current generation basically arise from the same GOx redox reaction. It has been reported that TTI can be used as a quality indicator when the difference in *E*a between the TTI and food is ± 20 kJ/mol [5]. Therefore, the biosenTTI could be used to monitor the quality of some foods such as frozen pork (*E*a = 35.5 kJ/mol), onions (*E*a = 35 kJ/mol), lettuce (*E*a = 37 kJ/mol), strawberries (*E*a = 39 kJ/mol), etc. [34,35].

**Figure 7:**
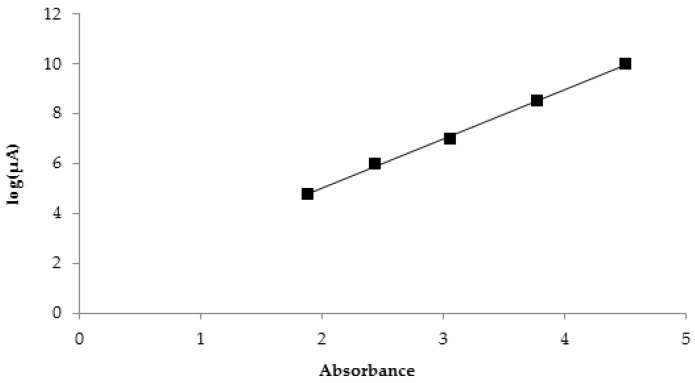
Correlation between the color change (abs) at 523 nm and the current generation at +1.3 V at 25 °C.

A linear correlation was observed between the colorimetric and electrical responses of biosenTTI (Figure 7). This is because they commonly followed a zero-order reaction. The correlation coefficient was 0.99. Consequently, it was confirmed that both the current output and the color changes work well as the TTI responses. 

The biosenTTI developed in this study generates a color change and current by a biochemical mechanism at the same time. So, it can be expressed as an electrical signal, rather than simply a visual change, compared with a conventional TTI [12,13,14,15]. Although it is still in the early stages of development, biosenTTI has the advantage of directly showing the change in the quality of food as a change, rather than simply integrating time-temperature history, as compared with LCD novel TTI. Unlike temperature data logger, biosenTTI has a function to provide directly the deterioration degree and remaining shelf life of foods other than time-temperature profile or history. 

TTI is usually used for chilled foods with short-term storage or distribution. As an example, chilled lettuce, an application of TTI, has the total distribution time of 72 h at the temperature of 3 to 17 °C. It is harvested at 15–17 °C, but it is cooled to 5 °C in a vacuum refrigerator after 3 h and then stored in a 5 °C storehouse for 5 h. The temperature fluctuates in the range from 3 to 15 °C during shipping. Lettuce is displayed at about 7 °C in retail stores such as Market [36]. In this case, the stability and performance of biosenTTI were assured because possibly unstable substance in use, GOx, can be stable for 213 days at 4–40 °C only by adjusting the TTI environments to optimal conditions of pH, enzyme stabilizer, and ionic strength [37].

## 4. Conclusions

Despite the longstanding recognition of TTI as an effective monitoring tool, its commercialization is still in the beginning stages. In this study, we aimed to improve the performance of the traditional form of TTI by integrating its function with a biosensor. Such a biosensor-type TTI could make it possible to provide digital information about food quality instead of analog information, like visible color. Throughout the tests, the GOx-based TTI functioned as a biosensor with significant current changes. This confirms the possibility that a conventional TTI could be transformed into a biosensor. In order to promote this prototype biosenTTI to a practical TTI, further attempts would be needed to adjust the *E*a values to the ranges more common in food applications, increase the enzyme stability, and produce the recognition and transducing elements in a test strip and a portable meter.

## Figures and Tables

**Table 1:** Comparison of reaction kinetics between TTI and TTI biosensor.

Parameters	TTI		BiosenTTI	
Temperature (°C)	*k (*1/min)	R^2 b^	*k (*µA/min)	R^2^
5	0.0187 ± 0.0005 ^a^	0.99	0.0360 ± 0.0020	0.99
15	0.0293 ± 0.0018	0.98	0.0566 ± 0.0026	0.99
25	0.0363 ± 0.0012	0.99	0.0716 ± 0.0024	0.99
35	0.0540 ± 0.0019	0.99	0.1073 ± 0.0028	0.99

^a^ True value with 95% confidence limit in linear regression analysis; ^b^ Coefficient of determination.
